# A Pilot Evaluation of WELLfed, a Community-Based Adult Education Intervention

**DOI:** 10.3390/ijerph22040526

**Published:** 2025-03-30

**Authors:** Kahurangi Jean Dey, Kankshita Dewan, Kim Murray, Donna Hiroki, Mona Jeffreys

**Affiliations:** 1 Te Hikuwai Rangahau Hauora|Health Services Research Centre, Te Herenga Waka-Victoria University of Wellington, Wellington 6011, New Zealand; kankshita.dewan@vuw.ac.nz (K.D.); mona.jeffreys@vuw.ac.nz (M.J.); 2 Te Kura Tātai Hauora|School of Health, Te Herenga Waka-Victoria University of Wellington, Wellington 6011, New Zealand; 3 WELLfed, Porirua 5024, New Zealand; kim.murray@wellfed.kiwi

**Keywords:** food security, community interventions, adult education, nutrition, self-efficacy, food insecurity

## Abstract

Introduction: WELLfed is a community-based adult education programme focused on improving food literacy, with a stated aim to “nourish communities through food and connections”, in a low-income community in Aotearoa New Zealand. Adult learners are taught food preparation and cooking skills in weekly interactive sessions. Here, we describe two pilot phases of a three-phase evaluation. Methods: Our overall aim is to evaluate the effectiveness of the WELLfed programme. Phase 1, in keeping with the co-design approach of WELLfed, co-designed outcome measures through five focus groups (*n* = 20) involving a wide range of stakeholders. Phase 2 developed and refined a survey based on these co-designed measures. The survey was administered to WELLfed learners at baseline (*n* = 15) and again towards the end of their programme (follow-up *n* = 12). Wilcoxon rank sum tests of significance were performed, restricted to participants with both baseline and follow-up data. Results: Five domains of importance identified in Phase 1 were as follows: (i) engagement support, (ii) food knowledge and skills, (iii) personal development, (iv) relationship transformation, and (v) community flourishing. Phase 2 findings include increased comfort level at engagement (*p* = 0.063). Food knowledge and preparation skills improved on a range of factors. All metrics of self-confidence and self-efficacy increased, with the greatest change being the ability to find support in times of crisis. Self-reported excellent or very good health increased (*p* = 0.050). Fewer people reported food running out due to a lack of money (*p* = 0.016) or eating less because of a lack of money (*p* = 0.008). Conclusions: The pilot study shows the effectiveness of the WELLfed programme for improving food knowledge and skills, reducing food insecurity, and improving health outcomes. Further research with a larger sample size is required to confirm these pilot results.

## 1. Introduction

Food insecurity, a lack of regular access to enough safe and nutritious food for normal growth, development, and an active and healthy life [1] is an increasing concern in Aotearoa New Zealand, highlighting experiences of hunger [2]. Driven by local factors such as inadequate income and high food prices, food insecurity disproportionately affects Indigenous Māori and Pacific peoples, and people in low-income households [3]. In the New Zealand Health Survey (NZHS), food insecurity is measured using eight standard questions, all focusing on affordability [3]. In the 2023/24 NZHS, 27% of children aged under 15 years reported living in a household where food runs out “sometimes” or “often”, a figure that was 34% for Indigenous Māori and 55% for Pacific children [4]. Each other metric of household food insecurity used in the NZHS, such as the use of food banks or food grants and eating less due to lack of money, shows similar inequities. Although the focus is on affordability, others have noted the importance of adequate quality as well as quantity of food, along with uncertainty around consistent access to food [5]. Food insecurity and nutritionally deficient diets are associated with undernutrition, missing essential nutrients, and multiple chronic health conditions such as diabetes, obesity, and heart disease.

Both government and not-for-profit organisations in Aotearoa New Zealand are involved in addressing food insecurity. Government initiatives to provide food assistance support include the New Zealand Food Network, a nationwide community food re-distribution network, Kore Hiakai, a zero-hunger collective and Aotearoa Food Rescue Alliance, a food rescue advocacy organisation [6]. Ka Ora, Ka Ako, the Healthy School Lunches Programme [7] and Healthy Families NZ, a systems-change initiative working at 11 locations across the country, [8] are other Government interventions. Common community-based activities include food banks, food rescues, social supermarkets, pātaka kai (community food pantries), fruit harvests, community gardens, and community meal initiatives [8].

Our research focuses on the WELLfed NZ Trust. Launched in 2016 as a pilot cooking class for parents at a local school, WELLfed is a registered New Zealand charity governed by a Board of Trustees. WELLfed is run by a CEO and a wider team of five full-time staff, 17 part-time staff, and 65 volunteers with an operating expenditure of less than NZD 500,000. Operating from a former bowling club site, WELLfed provides adult food literacy education and addresses food insecurity in a low-income area of Porirua, Greater Wellington [9]. Porirua has notably higher proportions of Māori and Pacific peoples and higher levels of deprivation than other areas in the Greater Wellington region. The programme has evolved through community co-design to ensure accessibility and cultural appropriateness and now primarily serves Māori and Pacific learners (92%). Over 800 classes have been conducted since inception. To support engagement, WELLfed provides learners with weekly fresh fruit and vegetables from a local food co-operative, kitchen equipment, and 25 basic pantry items. Beyond cooking, WELLfed offers practical, interactive personal development workshops, including First Aid, Interview Skills, and Communication Skills. The programme assists participants with obtaining a valid driver’s licence, reducing tension around law enforcement, and removing barriers to employment through a photo ID. Tutors, many of whom are past learners, lead the sessions, thus fostering a sense of community and giving visibility to personal development [9]. Community involvement is central to WELLfed’s approach, with 51% of the current team being programme graduates. The initiative extends to environmental stewardship through its garden programme, established in 2022. The garden serves as an education hub, a place for the community to volunteer and learn, and a source of fresh produce for the kitchen. It has hosted garden visits and harvested 1284 kg of food since being established. Additionally, a total of 27,150 kg of rescued food has been repurposed, diverting it from landfill for use in classes or safe distribution, further demonstrating WELLfed’s commitment to community flourishing and sustainable practices [9].

The 16-week interactive cooking course, held in WELLfed’s teaching kitchen, focuses on creating affordable meals (aiming for under NZD 10 per recipe) despite rising food costs. The curriculum covers meal planning, balancing food needs with available resources, food access, diet quality, and understanding the well-being impacts of healthy eating, aligning with nutritional recommendations for Aotearoa New Zealand. Each session includes a demonstration, individual meal preparation, discussions about food and kitchen safety practices, and culminates in a communal meal of the foods prepared that session. Learners are taught food preparation skills, focusing on healthy, nutritious, low-cost family meals with seasonal fruit and vegetables and items commonly included in food parcels. The hands-on approach to nutrition education and cooking is an effective means of teaching adult learners [10]. Informal evaluation, using both qualitative and quantitative data, suggests WELLfed has significant beneficial impacts for food-insecure households in a low-income community: reported outcomes include learners’ increased confidence, healthier eating habits, and spending less on food following the course [11].

## 2. Literature Review

Low-income and food-insecure populations are shown to have poor dietary behaviours, limited food preparation skills, and low levels of self-efficacy toward preparing healthy meals [12]. Urbina [13] argues an erosion in self-efficacy and food literacy has led to a reliance on prepackaged foods and eating foods outside the home that are often high in fat, salt and calories, leading to deterioration in diet quality and an increase in non-communicable diseases. She reviews the ‘Moms in the Kitchen’ cooking programme in terms of four dimensions of food literacy—self-efficacy, behaviour, knowledge, and empowerment [13]. Self-efficacy, behaviour, and knowledge incorporate the ability to plan and manage purchasing and intake, select quality foods and prepare tasty meals from available foods, understand the impact of food on well-being, and be able to eat in a social way [14]. Empowerment is the ability to make changes for optimal food consumption. Food quality is as important as cooking skills, particularly cooking with minimally processed foods when cooking at home [15]. Adults with poor cooking skills and adults who cook with highly processed foods were less likely to have adequate fruit and vegetable intake and very good general health or mental health compared to those with advanced cooking skills and those cooking with minimally processed foods. Interestingly, adults with poor cooking skills were less likely to have obesity than those with advanced cooking skills, but adults who cooked with highly processed foods were more likely to have obesity than those who cooked with minimally processed foods. Interventions that promote improving cooking skills and cooking with minimally processed foods have the potential to promote better health and diet outcomes [15]. In a pilot community intervention, a one-hour presentation involving a cooking demonstration, taste testing, basic education on a healthy food guide (MyPlate), and basic food safety showed no significant improvements in participants’ confidence to prepare healthy meals but did result in significant gains in knowledge related to healthy food guidelines and food safety [12]. Driver and Friesen [12] identify a gap in strategies for translating improved nutrition knowledge into improved self-efficacy and behaviour change—improvements in cooking skills require interventions designed around practising cooking.

## 3. Sociotype Conceptual Framework

Sociotype is a concept useful for understanding factors in addition to bio-medical pathways that influence an individual’s growth, development, and behaviour and “for understanding how people manage life in general, and chronic disease in particular” [16]. A person is “the product of [their] genotype, phenotype and sociotype” [17]. Berry and De Geest [16] develop sociotype into a conceptual framework, consisting of three domains of influence: individual (intra-personal), relationships (inter-personal), and environment (wider context):

“… nutrition in its widest sense has a major influence on the development of the sociotype throughout the life cycle. This was anticipated at the *individual* level by Brillat-Savarin (1755–1826) in his well-known saying: “Tell me what you eat, and I will tell you what you are.” We may broaden this construct to the level of *relationships*: “Tell me how a family eats, and I will tell you how it functions.” And finally, the extension of sociotypic analysis and nutrition to the level of the *environment*: “Tell me how a nation eats, and I will tell you its values.” Do, for example, children go to bed hungry? (Food security). The sociotype determines how an individual adjusts to life in general and disease in particular” (italics in original) [16].

The interactions between the three domains can be useful in the consideration of food insecurity in broader contexts [18]. The sociotype conceptual framework has been used to contextualise a variety of situations, including food insecurity and global challenges due to climate change [18], food insecurity during the COVID-19 pandemic [19], food insecurity in households with children with autism spectrum disorders and intellectual disabilities in the United States [20], and the affordability of healthy diets compared to habitual diets in Australia [21]. Applying the sociotype framework highlights opportunities for intervention along pathways at each level such as welfare safety-net safeguarding, school food programmes, and food and nutrition education [21].

## 4. Aims of This Study

Although there are many charitable and other non-governmental initiatives to address food insecurity, few are evaluated and, hence, rarely expanded beyond their original community or district [10]. This presents a gap in both the literature and practice. The overall aim of the research is to determine the effectiveness of a community-based adult education intervention at increasing food literacy, improving confidence in preparing healthy family meals using limited resources, and addressing food insecurity for households in a low-income community. To achieve the aim, the two objectives of the study are as follows: (i) to co-design community-relevant outcome measures and (ii) to develop and refine a survey for pilot data collection on these co-designed domains. A second aim will provide estimates of the magnitude of change that WELLfed sessions can achieve, to inform sample size calculations for the third phase of the research programme, which will involve a full formal evaluation.

## 5. Methods

We undertook a mixed-methods, two-phase approach to the evaluation. Phase 1 involved stakeholder focus groups to co-design outcome measures with the WELLfed community. Phase 2 involved developing and refining a survey based on the co-designed outcome measures, followed by surveying a cohort study of new learners. The population for this pilot study included a convenience sample of WELLfed stakeholders for Phase 1 (*n* = 20). For Phase 2, the target population was new learners at WELLfed, and the study population comprised those who were available on a day that the Research Assistant was able to assist learners in completing the survey (*n* = 15). Inclusion criteria for both phases required that participants be at least 18 years of age. Forms and processes were designed to be inclusive for participants with low literacy levels. All participants received a supermarket voucher as consideration for their time and contribution to the research. The research team selected the WELLfed premises in Porirua as the most appropriate site for the data collection because participants know it as a safe space. Times were selected that were convenient for participants, for example, the afternoon the tutors did not teach a class. One of the key challenges with the focus groups was to elicit people’s genuinely held views, so significant time was spent at the start of each focus group on whakawhanaungatanga (relationship building) to develop trust. Ethics approval for both phases was obtained from the Human Ethics Committee of Te Herenga Waka–Victoria University of Wellington prior to the research phase starting.

## 6. Data Collection Phase 1 Focus Groups

For Phase 1, from September 2023 to January 2024, the research team spoke with 20 WELLfed stakeholders in five focus group settings. Focus groups were preferred over other data collection methods because they are collaborative processes by design and consistent with the round-the-table style of learning environment at WELLfed. Focus groups allowed people to speak up when they felt comfortable and also to remain silent. The focus groups included five tutors; three current or recent WELLfed learners; four current or recent Māori learners in the kaupapa Māori [22] session; six wider stakeholders, including garden and Next Steps staff, past and present volunteers; and two board members (*n* = 20). Following WELLfed’s sensitivity to research processes, no demographic data were requested from the focus group participants. Written information was provided, but also explained verbally for people with low levels of literacy, and informed consent was obtained from each participant. We used a conversation guide for all focus groups, which included questions to initiate conversations around why WELLfed works or does not work and, hence, what measures would be important to include in a formal evaluation of WELLfed. Two or three researchers were present at each focus group; one researcher was nominated to lead, and the others observed, took notes, and joined the discussion as appropriate. The discussions were audio-recorded to allow for fact-checking but not transcribed for formal thematic or other qualitative analysis. We identified five domains of influence of WELLfed, broadly mapped to the stages that learners experience through their WELLfed journey: (i) engagement support; (ii) food knowledge and skills; (iii) personal development; (iv) relationship transformation; and (v) community flourishing.

## 7. Data Collection Phase 2 Survey

Phase 2, from March 2024 to September 2024, involved quantitative measurement of markers in each domain of influence. A survey was developed that included topic questions to address each domain—Engagement Support, Food Knowledge and Skills, Personal Development, Relationship Transformation, and Community Flourishing. An additional topic of Food Security was included for comparison with questions specifically asked in the New Zealand Health Survey. Survey questions were identified from existing question banks, prioritising questions that have been used in surveys in Aotearoa New Zealand. Specific surveys used included the New Zealand Attitudes and Values Study [23], the General Social Survey [24], Te Kupenga [25], and the Programme for International Student Assessment [26]. We also included food insecurity and general health questions from the New Zealand Health Survey (NZHS) [27].

A survey tool of 46 questions was finalised after testing with a previous WELLfed learner. The response options to most survey questions were displayed graphically using a five-point Likert-equivalent scale of faces, ranging from a green, smiling face to a red, angry face (see Supplementary Materials). The food insecurity questions had responses used in the NZHS: “often”, “sometimes”, or “never”. Five socio-demographic questions (age, gender, ethnicity and household composition) were collected, as in the 2023 New Zealand Census [28]. The baseline survey was administered face-to-face with 15 new learners (*n* = 15). After the first six surveys were administered, the questions were further refined and reduced from 46 to 39 questions, following feedback from participants and the research assistant who administered the surveys. Wave 1 of the survey was administered either just before or within the first month of starting at WELLfed. All participants received a copy of the survey, but the questions were also read out loud by the research assistant and explained if needed.

The same survey was re-administered near the last week of each participant’s time at WELLfed (Wave 2), about 16 weeks later. This was carried out either face-to-face or over the phone if the participant preferred. Three of the survey participants were unable to be contacted at the end of the intervention, so the follow-up survey was conducted with 12 people (*n* = 12). Data were entered into an Excel spreadsheet, with answers coded as 1 to 5 according to the Likert scale. Data were imported to Stata, version 17, for statistical analyses [29]. Outcomes were dichotomised to compare people in the upper two categories with the lower three categories (see table footnotes for descriptions of the categories, which varied by outcome measure). Average responses for each survey wave were calculated and described. For each question, the within-person change from the first to the second wave was calculated, and the average change across respondents was described. Wilcoxon rank sum tests of significance were performed, restricted to those people who had both baseline and follow-up data. Median and interquartile range (IQR) of change, and two-sided exact *p*-values are reported.

## 8. Results

Following WELLfed’s sensitivity to research processes, no demographic data were requested from focus group participants. All of the survey respondents were female (100%); most were Māori (*n* = 13, 81%) and included people from all age groups, although the most common (*n* = 6, 40%) was 25 to 44 years. Most survey participants had health challenges; only four (27%) reported excellent or very good health at the baseline survey. In this section, we summarise the five key domains identified that make WELLfed a success: engagement support, food knowledge and skills, personal development, relationship transformation, and community flourishing.

## 9. Engagement Support

This domain encompasses comments relating to a welcoming and non-judgemental atmosphere where learners can work at their own pace. It speaks to the importance of feeling accepted, respected, and heard. Understanding, kindness, and support were key components, as were facilitating everyone’s ability to learn and grow. Focus group participants spoke at length about WELLfed being a safe and trusted place. The people in the focus group of WELLfed tutors and the CEO spoke about creating a warm and welcoming environment where new starters felt they belonged. Some learners in the kaupapa Māori focus group noted that WELLfed feels like being on a marae (Māori cultural complex). Providing an environment that supports confidence and growth was central to this domain.

The survey results suggested that feeling comfortable or very comfortable coming into WELLfed increased from baseline to follow-up (*p* = 0.063). Likewise, feeling part of the WELLfed whānau (wider family) increased, though non-significantly, from baseline to follow-up (*p* = 0.13). At baseline, 87% (*n* = 13) agreed or strongly agreed that it was a place that feels like a marae (Māori cultural complex) or a place where they belong; in the follow-up survey, all participants strongly agreed with this (*p* = 0.25).

## 10. Food Knowledge and Skills

This domain focuses on competence in food preparation, cooking, and shopping. Topics that were raised included understanding food and nutrition, learning about allergies, growing your own food, and making healthier food choices, as well as cost-saving measures such as bulk buying, using grocery apps to find cheaper options, and minimising food waste. Cooking and eating together, along with the happiness it can bring, were highlighted. Confidence building around healthful food was a significant aspect of this domain.

The quantitative data supported the hypothesis that WELLfed increases people’s confidence in cooking and providing meals. In the baseline survey, the proportion of respondents who were “very confident” or “confident” (the upper two categories on the visual Likert scale) in any of the areas asked ranged from 33% to 67%; at follow-up, this ranged from 75% to 100% (see Table 1). Reported confidence was lowest at baseline for being able to make meals at home that are affordable (33%), which had increased to 92% at the follow-up survey.

WELLfed also had an impact on increasing the proportion of people who cooked at home and reducing how often they ate takeout meals. At baseline, two people (14%) cooked at home five or more times a week; at the follow-up survey, this had increased to five of the 12 respondents (42%), *p* = 0.12. At baseline, six people (40%) had takeout meals three or more times a week; at follow-up, this had reduced to two people (16%), *p* = 0.031.

## 11. Personal Development

This domain revolves around the confidence and motivation to acquire other skills outside of cooking. These include practical skills such as budgeting and literacy, as well as others like goal setting and public speaking. The emphasis was on learning by doing, with achievements celebrated and knowledge gained, leading to increased self-confidence and self-belief.

The survey data showed that WELLfed had an impact on self-reported self-confidence and self-efficacy. At baseline, these metrics were reasonably high, with the lowest areas being setting goals and feeling able to cope in difficult situations. At the follow-up survey, all metrics had increased, with the greatest change being the ability to find support in times of crisis. Further details are shown in Table 2.

## 12. Relationship Transformation

This domain identifies the importance of initiating and making connections, feeling part of a shared experience, and, for some learners, healing from past trauma. Being part of a collective seeking wellness was identified as important. The domain emphasises family involvement, from positive feedback on recipes, teaching kids to cook, eating together, and sharing experiences. The power of food to bring people together, create conversations, and forge stronger bonds within families was underscored. The ripple effect of the participant’s actions and their own learning, with knowledge and skills being passed on within families, leading to overall whānau (families) flourishing, is core to this domain.

The quantitative data showed increases in the measures of social connection, although none of these reached statistical significance. In the baseline survey, nine people (60%) reported that their whānau (family) was doing very well or well; this had increased to 83% (10 people) at follow-up, with a median improvement of 1.5 (IQR: 0 to 1.5) on the 5-point scale (*p* = 0.15). At baseline, 10 people (75%) reported that their whānau (family) got on well or very well with one another. This was slightly higher at follow-up (10 people, 83%), but no difference in the change analysis (*p* = 0.60). Feeling connected to groups in their community was reported by 10 out of 13 people (77%) at baseline and nine out of 12 people (75%) at follow-up, with a small, non-significant median increase (0.5 points, IQR: −1 to 2, *p* = 0.64) among the 10 people who completed both surveys.

## 13. Community Flourishing

This domain captures the broader impact of the programme, with examples given, including cooking using WELLfed recipes at marae (Māori cultural complexes) and the sharing of meals at community events. The ripple effect of the organisation’s actions in the wider community, leading to overall community flourishing, is central to this domain.

All respondents said that they had talked with whānau (family) or friends about WELLfed, and all the examples that were given of the topics they talked about were positive. Being able to provide food for occasions and events is particularly significant in Indigenous Māori and Pacific communities. People praised the outreach and impact of WELLfed, with more than one saying WELLfed changed lives in the community. Self-reported health increased markedly. At baseline, four people (27%) reported excellent or very good health, which had increased to six people (60%) at follow-up, with a median increase of 1.5 (IQR: 0 to 2) points on the five-point scale (*p* = 0.050).

## 14. Food Insecurity

There are eight metrics used to quantify different aspects of food security. Overall, there was a high degree of food insecurity at baseline, with no participants reporting that they “often” could afford to eat properly, and high proportions who experienced running out of food, eating less because of a lack of money, and relying on others or on food banks—all contributing to high levels of stress (see Table 3).

There was strong evidence that WELLfed reduced how commonly people reported food running out due to a lack of money (a change from 87% to 50%, *p* = 0.016) or eating less because of a lack of money (87% to 33%, *p* = 0.008). All other metrics indicated that WELLfed had a beneficial effect, except for a non-significant increase in people reporting limits in the variety of foods they eat and the use of food banks.

## 15. Discussion

The programme WELLfed, a nutrition-focused community-based adult education programme in the Porirua region of greater Wellington, has demonstrated significant positive impacts across multiple domains. Our findings indicate that WELLfed is influential in several critical ways beyond improving food and nutrition knowledge. The safe learning environment at WELLfed was identified as facilitating positive outcomes. Participants showed marked improvement in personal development skills, such as self-confidence and self-efficacy, supporting the programme’s value for employment skills development. The effects on the whānau (family) and community highlight that the benefits of WELLfed extend to the wider community. While limited in sample size and not powered to detect differences, many of the metrics used to operationalise the identified domains of influence demonstrated statistically significant results.

At the individual sociotype level, we have demonstrated that the sense of belonging that people felt, even in the baseline survey, indicates the importance that WELLfed staff place on making each person feel welcome, even before their course starts. Facilitators to initial attendance in an experiential cooking programme identified by Urbina [13], such as recruitment from trusted professionals, time for self-improvement, and support to change diet, are all evident at WELLfed. These facilitators to initial attendance, and other facilitators to continued attendance, such as trying new foods at no personal cost and low risk, having regular weekly classes at the same time, and classes being pitched at the right level with appropriate course length—not too long, but long enough to proceed at a good pace [13], should all be considered in the full evaluation. Given that engagement support was identified in the domains as one of the main attributes of WELLfed, it is important that it is recognised in other interventions as being central to their successful implementation.

Co-design is a feature of the WELLfed approach, where they collaborate with the local community to make the classes accessible and ensure food utilisation is appropriate and relevant to food needs in a low socioeconomic community. Our approach to the evaluation and pilot data collection used the same kaupapa (ethos), which is a significant strength of this research. The co-design approach is used in social return on investment methodology [30], where the wider impacts of interventions are identified and measured beyond those that a particular intervention may be designed to address. Throughout the focus groups, participants spoke about the features that evolved from co-design, such as interactive cooking and eating together, and the holistic support features, such as childcare. Our survey results show participants valued their learning experience, increasing in confidence across a range of kitchen activities. WELLfed activities consider the individual as part of a family system; participants learn to cook to improve the health of their children and appreciate the programme operating during school hours, as well as the volunteers who mind preschool children during class times.

One food and nutrition security intervention in Aotearoa that has been formally evaluated is Ka Ora, Ka Ako, the government-funded free school lunch programme [31]. That evaluation found reported beneficial impacts on food security, equity, nutritional knowledge, well-being, and reduced financial hardship. Healthy eating initiatives need to address the interplay of different factors that are behind food insecurity. To be effective, for instance, healthy eating initiatives must focus on making nutritious food affordable, but in a way that culturally aligns with the recipients [32]. One of the negative impacts of Ka Ora, Ka Ako was the loss of agency for some students, who had been used to having choices about what and when they ate [31]. The benefit of education, as well as food provision, is a key strength of WELLfed to break the intergenerational cycle of food insecurity and/or reliance on packaged food. A nutrition and culinary education intervention for college students found a beneficial effect on measures of food insecurity [33]. However, college students are at a significant advantage in navigating the food insecurity landscape at the sociotype individual level, compared to WELLfed learners, many of whom have low literacy levels, and live in a suburb with no supermarket.

There has been no significant progress in Aotearoa New Zealand since the early 2010s on the implementation of policies and infrastructure support for healthy food environments [34], which is reflected in the lack of improvement and, in some cases, the worsening of food insecurity metrics [4]. On a household level, a randomised controlled trial of the provision of supermarket vouchers to food-insecure households increased spending on food, concluding that direct food assistance for low-income households via supermarket vouchers increased overall household food expenditure [35]. However, as noted above, ensuring food security for generations to come requires more than food assistance. A national Food Security Strategy would confirm the government’s commitment to addressing food security aspects, including availability, accessibility, utilisation, and stability; the food system requires urgent action to protect the health and well-being of the public [36]. In the absence of effective national coordination, programmes like WELLfed play a crucial role in improving household food security at the local level. Other local food assistance initiatives such as community gardens, community-supported agriculture, farmers’ markets, food banks, and local food networks have the potential to provide both immediate food relief and long-term food resilience based on their execution. However, caution is needed in assuming all local food initiatives inherently improve food access and security [23,26]. An institutionalised and increasingly corporatised charitable food assistance system reinforces the notion that the charitable system is an effective way to address hunger and improve food security, ultimately relieving governments of responsibility to take action and address upstream drivers of food insecurity through adequate financial and social supports [37]. Issues of inequity remain unaddressed in some popular alternative food programmes, for example, where a ‘greater good’ framing reinforces experiences of exclusion and alienation, reproducing inequality and marginalisation for food-insecure community members [38]. Regardless of where they are grown, the increasing costs of fruit and vegetables are a deterrent to their inclusion in regular diets for socioeconomically disadvantaged households [32]. The COVID-19 pandemic and the current cost-of-living crisis have profoundly altered the job market, housing, and living expenses, worsening existing challenges to access affordable, healthy food [39], and exacerbating entrenched inequities.

Poor diet and low income contribute to a double dose of preventable health burden for Māori in Aotearoa: higher rates and worse outcomes [40]. The senior researchers on the study (KD and MJ) worked together in a way underpinned by the Te Tiriti o Waitangi (Treaty of Waitangi) Relationship Framework [41], ensuring that the voice of Māori was central to all decision-making surrounding the design, analysis, and interpretation of the results. Employing a Māori former learner and current WELLfed tutor (DH) as a research team member proved invaluable. DH’s lived experience was explicitly valued and heard, and her involvement proved critical at several stages throughout the research. Having an insider researcher established trust and credibility during the focus groups. DH’s insights about the Likert-equivalent survey were crucial in developing a survey tool suitable for a community with low literacy levels. The authenticity and integrity of DH’s contributions led to improved data collection, particularly for sensitive food insecurity questions relating to lack of income and decision-making around limited eating. Māori participation throughout the work recognises that the impact of food insecurity reaches beyond the individual into their whānau (families) and communities, and makes Māori perspectives visible throughout the inquiry, strengthening the results.

The study is limited by the small number of survey participants (*n* = 15) and loss to follow-up (*n* = 3). These two phases of the work were designed as a pilot. However, due to a lack of ongoing funding for the research, we thought it was important to publish the results—a decision that was made prior to the results being analysed. A further limitation is the potential bias introduced by the three participants lost to follow-up. Loss of participants could potentially skew results, either positively (if their outcomes were less favourable than those who remained engaged, thus artificially inflating the reported benefit) or negatively (if they had moved on to full-time work or study, as some WELLfed learners do).

In summary, we report good evidence of the beneficial effects of WELLfed across a wide range of domains, extending beyond nutrition education to impact self-efficacy, community well-being, and potentially mental health. While this pilot study has limitations, it provides a strong foundation for a future, more comprehensive evaluation to confirm definitive results. Planning for this work is underway and aims to include the following: a larger sample size; improved retention strategies, including a greater effort to keep in touch with all learners to avoid low numbers to follow-up; Kaupapa Māori analysis [22]; Pacific-specific analysis; social return on investment measures [30]; and explicit measures of mental health and well-being outcomes. The success of WELLfed illuminates the importance of community-led, culturally appropriate initiatives to alleviate the impacts of household food insecurity at the local level. However, it also highlights the need for broader policy and strategy approaches to address poverty and the root causes of food insecurity in Aotearoa New Zealand.

## Figures and Tables

**Table 1:** Confidence in various kitchen activities in the baseline and follow-up surveys.

	Baseline (*n* = 15)	Follow-Up (*n* = 12)	Change * (*n* = 12)	*p*-Value
How confident are you in …?				
using measuring spoons/cups	10 (67%)	12 (100%)	1 (0 to 2)	0.016
boiling an egg **	6 (67%)	11 (92%)	0 (0 to 1)	0.50
using a slow cooker	6 (40%)	10 (83%)	1 (0.5 to 2)	0.014
following a written recipe step-by-step	10 (67%)	12 (100%)	1 (0 to 1.5)	0.016
trying new recipes with vegetables	9 (60%)	12 (100%)	1 (0 to 2)	0.008
trying new recipes with canned foods	6 (40%)	9 (75%)	1 (0 to 3)	0.031
using fresh herbs (such as parsley) and spices	7 (47%)	11 (92%)	1.5 (0.5 to 3)	0.004
baking	9 (60%)	10 (83%)	1 (0 to 1.5)	0.008
making meals at home that are affordable	5 (33%)	11 (92%)	1.5 (0.5 to 2)	0.004
making meals at home that are healthy	6 (40%)	10 (83%)	1.5 (1 to 3)	0.001

Table 1 shows the number and proportion of people who were confident or very confident in various kitchen activities in the baseline and follow-up surveys. * Median (interquartile range) of intra-individual change on a 5-point Likert scale, with a positive change indicating an increase in confidence. ** This question was added after the first six participants had completed the survey, so it was only answered by nine people. The first six participants are excluded from the denominator of the baseline survey respondents.

**Table 2:** Measures of self-confidence and self-efficacy in the baseline and follow-up surveys.

	Baseline (*n* = 15)	Follow-Up (*n* = 12)	Median Change * (*n* = 12)	*p*-Value
I can learn almost anything if I set my mind to it	12 (80%)	12 (100%)	0.5 (0 to 1)	0.031
I usually have control over the way my life turns out	7 (50%) †	9 (75%)	1 (0 to 1)	0.12
I feel proud that I have accomplished things	12 (80%)	12 (100%)	0 (0 to 1)	0.063
I feel that I can handle many things at a time	7 (47%)	10 (83%)	1 (0 to 2)	0.13
I feel that I can cope when I’m in a difficult situation	6 (40%)	8 (67%)	0.5 (−0.5 to 1)	0.38
I can easily find support if I need it, for example to make appointments	11 (73%)	12 (100%)	1 (0 to 1.5)	0.050
I can easily find support in times of need or crisis	8 (53%)	12 (100%)	1.5 (0 to 2.5)	0.016

Table 2 shows the number and proportion of people who agreed or strongly agreed with the statements shown and the median change in score from baseline to follow-up, with strongly agreed coded as 1 and strongly disagreed coded as 5. * Median (interquartile range) of intra-individual change on a 5-point Likert scale, with a positive change indicating an increase in agreement. † Based on 14 people.

**Table 3:** Food insecurity in the baseline and follow-up surveys.

	Baseline (*n* = 15)	Follow-Up (*n* = 12)	Median Change (*n* = 12)	*p*-Value
We can afford to eat properly *	14 (100%)	10 (91%)	0 (0 to 1)	0.69
Food runs out in our household due to a lack of money	13 (87%)	6 (50%)	1 (0 to 1)	0.016
We eat less because of a lack of money	13 (87%)	4 (33%)	1 (0 to 1)	0.008
The variety of foods we are able to eat is limited by a lack of money	3 (38%)	7 (58%)	0 (−1 to 0)	>0.99
We rely on others to provide food and/or money for food when we do not have enough money for food	8 (62%)	5 (42%)	0 (0 to 1)	0.25
We make use of special food grants or food banks when we do not have enough money for food	9 (69%)	9 (75%)	0 (0 to 0)	>0.99
I feel stressed because of not having enough money for food	9 (80%)	6 (50%)	0 (0 to 1)	0.41
I feel stressed because I can’t provide the food I want for social occasions	11 (79%)	4 (33%)	0 (0 to 2)	0.094

Table 3 shows the number and proportion of people who stated that they faced the stated food insecurity metric “often” or “sometimes”. The median change is calculated across three categories (often, sometimes, never), with a positive change indicating better food security at follow-up. People who did not answer the question or stated “don’t know” or “prefer not to say” were excluded from the analysis. * Due to the wording of the question, the reported proportion is for people who answered “sometimes” or “never”.

## Data Availability

The datasets presented in this article are not available because consent for sharing of data was not obtained from participants.

## Supplementary Materials

The following supporting information can be downloaded at: https://www.mdpi.com/article/10.3390/ijerph22040526/s1, Final Version of the Survey.
