# Supplementary material for: A Pilot Evaluation of WELLfed, a Community-Based Adult Education Intervention
Source: Int J Environ Res Public Health. 2025 Mar 30;22(4):526. doi: 10.3390/ijerph22040526 (PMC12027199; doi:10.3390/ijerph22040526)
Supplement: Supplementary file 1 [file ijerph-22-00526-s001.zip › ijerph-3495533-supplementary.pdf]

Feeling part of WELLfed

Circle the picture that best describes your answer.

1. How long you been coming to WELLfed?
   - ☐ New learner / I haven’t yet started
   - ☐ Less than a month
   - ☐ 1-2 months
   - ☐ 3-6 months
   - ☐ More than 6 months

2. Coming into WELLfed, how comfortable do you feel in this space?

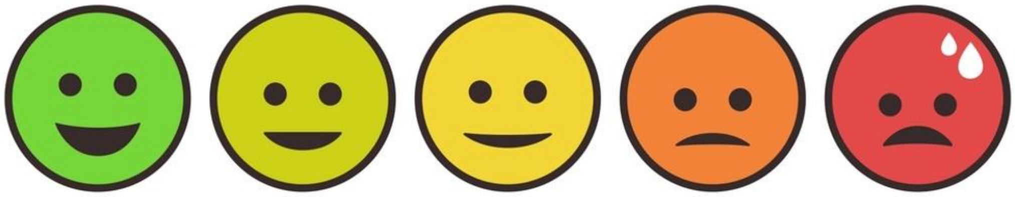

3. How much do you feel part of the WELLfed whānau?

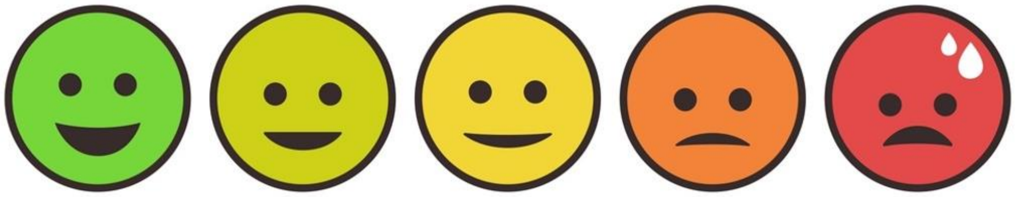

4. How much does WELLfed feel like a marae / a place where you belong?

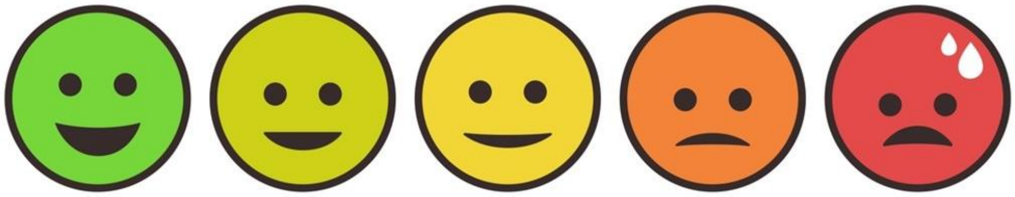

Food competence, nutrition knowledge and skills

Circle the picture that best describes your answer.

5. How confident are you in using measuring spoons/ cups?

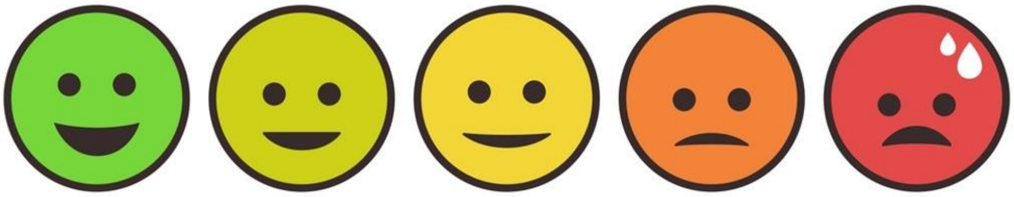

6. How confident are you in boiling an egg?

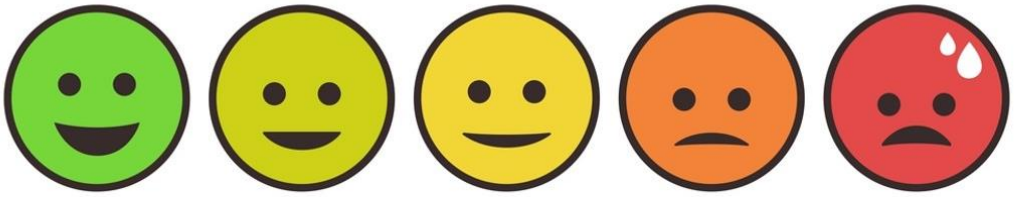

7. How confident are you in using a slow cooker?

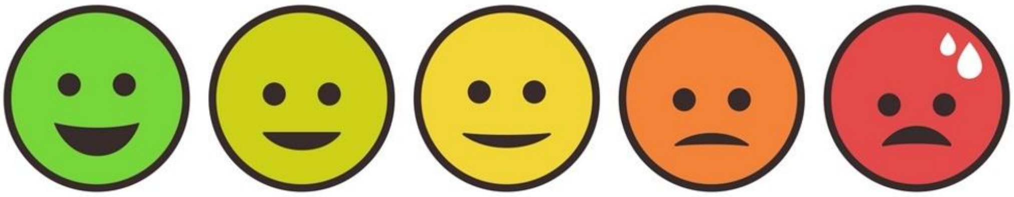

8. How confident are you in following a written recipe step-by-step ?

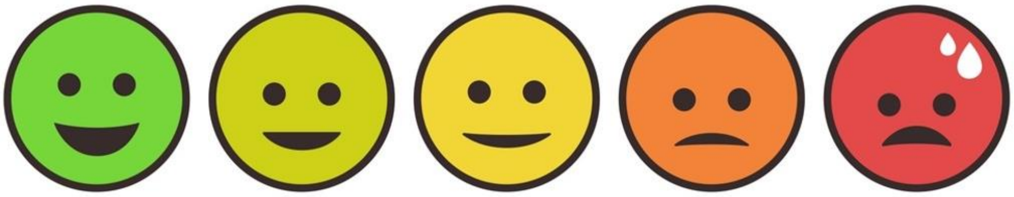

9. How confident are you in trying new recipes with vegetables?

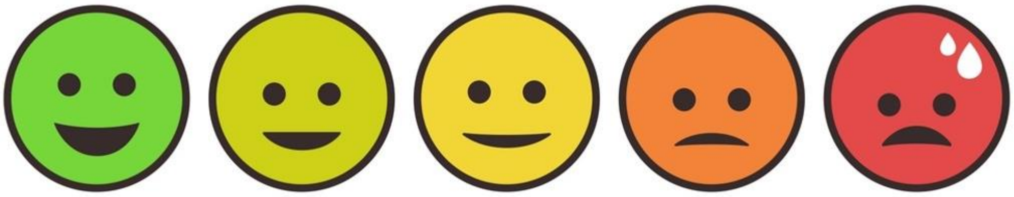

10. How confident are you in trying new recipes with canned foods?

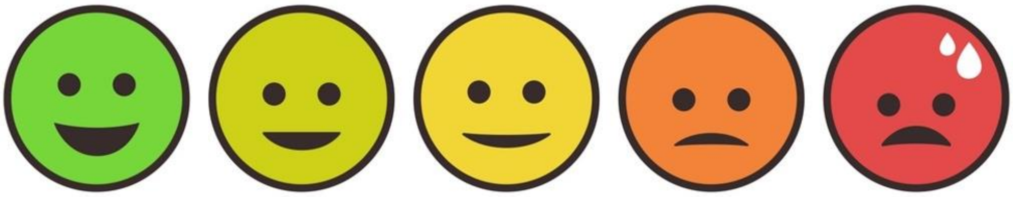

11. How confident are you in using fresh herbs (such as parsley) and spices in cooking?

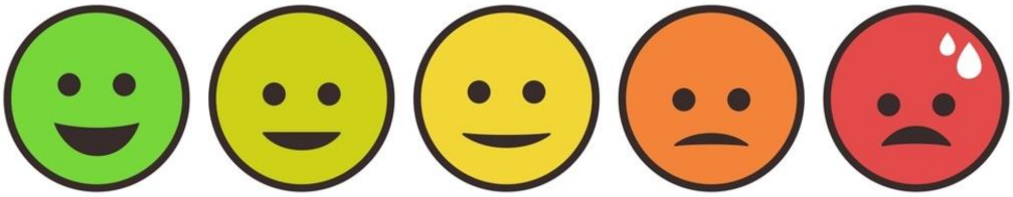

12. How confident are you in baking?

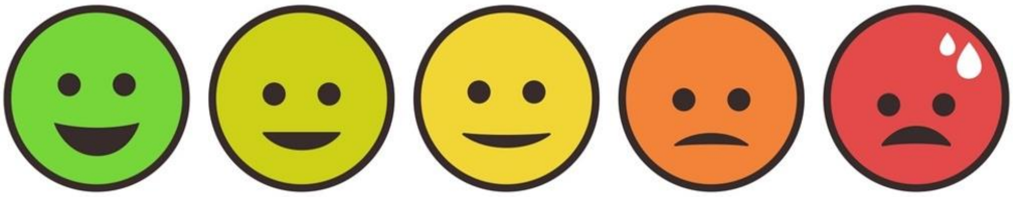

13. How confident are you in making meals at home that are affordable?

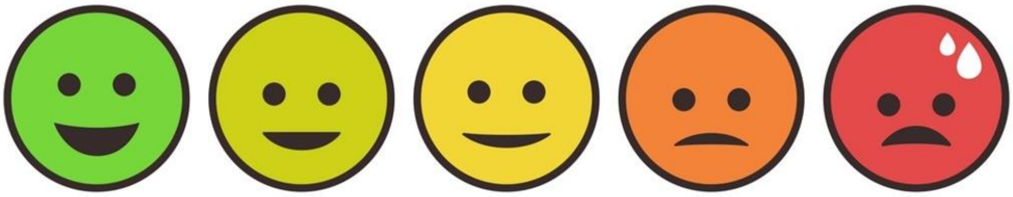

14. How confident are you in making meals at home that are healthy?

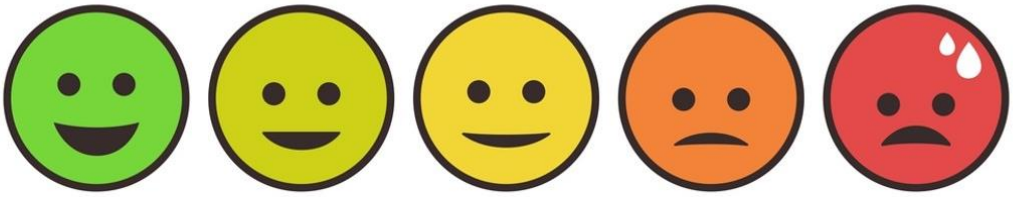

Self-confidence, personal skills development, empowerment and self-efficacy

Circle the picture that best describes your answer.

15. I can learn almost anything if I set my mind to it

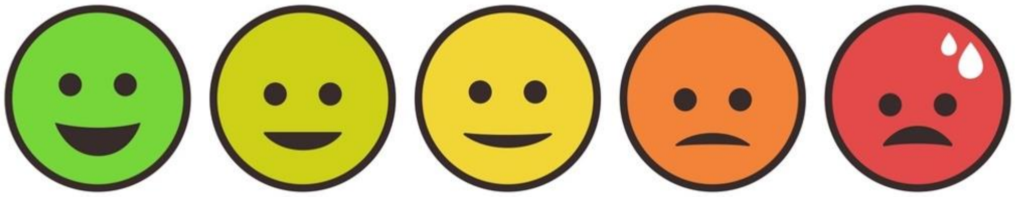

16. I usually do not set goals

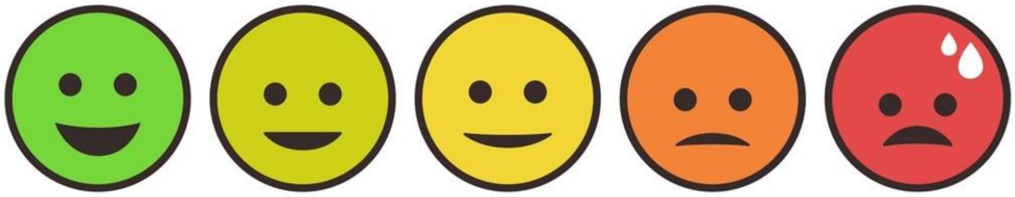

17. I usually have control over the way my life turns out

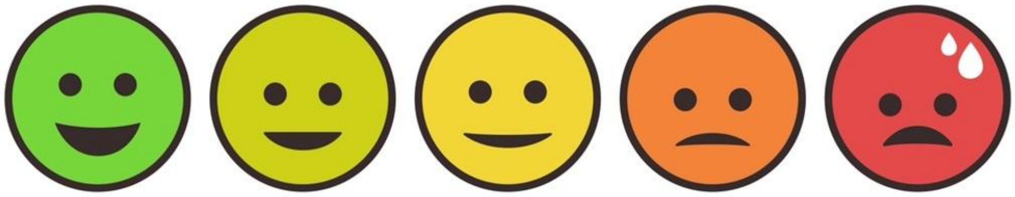

18. I feel proud that I have accomplished things

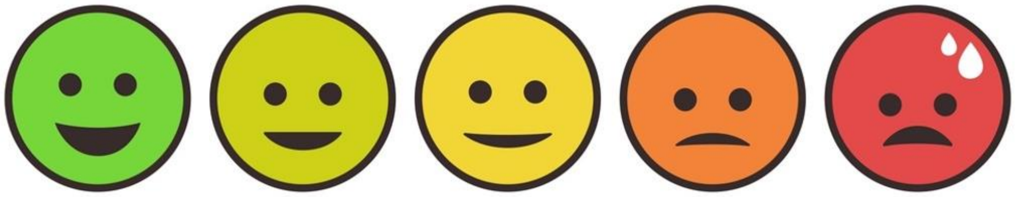

19. I feel that I can handle many things at a time

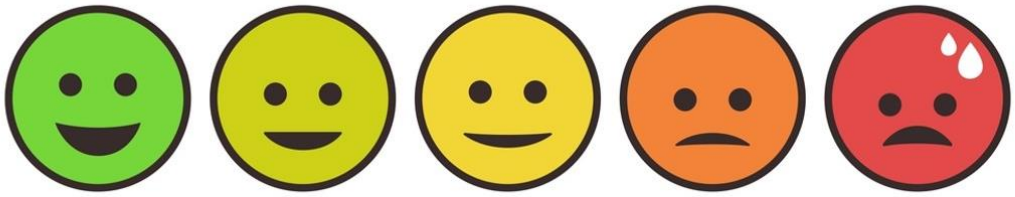

20. I feel that I can cope when I'm in a difficult situation

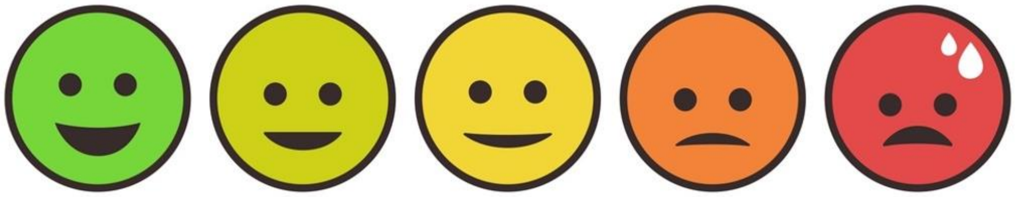

21. I can easily find support if I need it, for example to make appointments

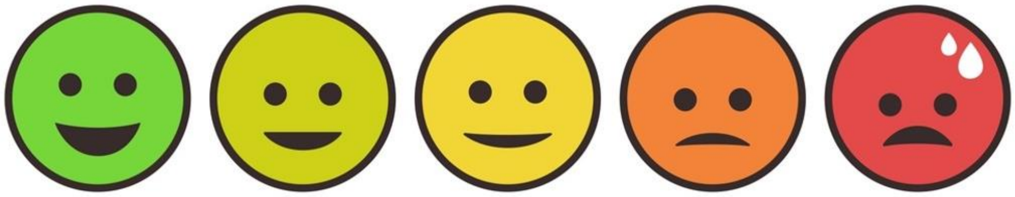

22. I can easily find support in times of need or crisis

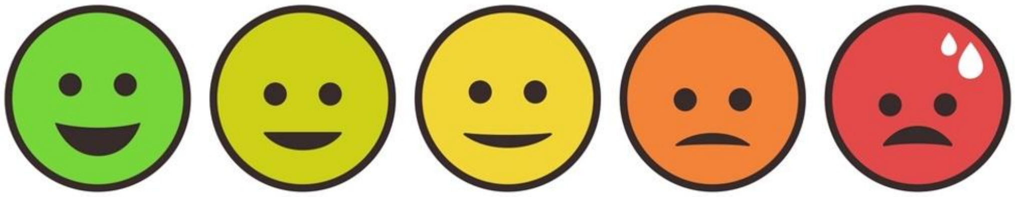

Social connection (learners in the community)

Circle the picture that best describes your answer.

23. How well is your whānau/ family doing?

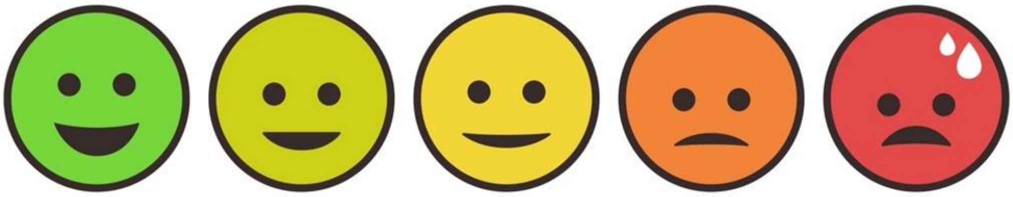

24. How well do your whānau/family get along with one another?

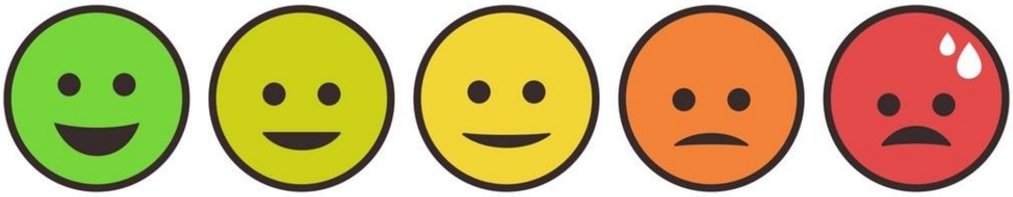

25. How much do you feel a part of groups in your community?

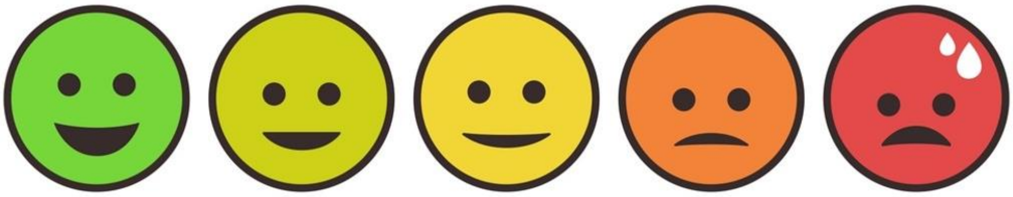

26. During the last week, how often did you cook at home?

☐ Not at all
☐ 1-2 times
☐ 3-4 times
☐ 5-6 times
☐ Every day

27. During the last week, how often did you have takeaways or eat out?

☐ Not at all
☐ 1-2 times
☐ 3-4 times
☐ 5-6 times
☐ Every day

Outreach and impact (WELLfed in the community)

28. Have you grown your own fruit or vegetables in the last 12 months?

- ☐ Yes
- ☐ No

  a. If yes, who with? (tick all which apply)

  - ☐ By self
  - ☐ With whānau
  - ☐ With WELLfed
  - ☐ With iwi, hapū, marae
  - ☐ With friends, neighbours, a local community or church group
  - ☐ Other, please say who……………………….

29. Have you ever talked with friends or whānau members about WELLfed?

- ☐ Yes
- ☐ No
- ☐ Not sure

  b. If yes, what sort of things have you talked about?

……
……
……
……

30. How likely are you to recommend WELLfed to your family and friends?

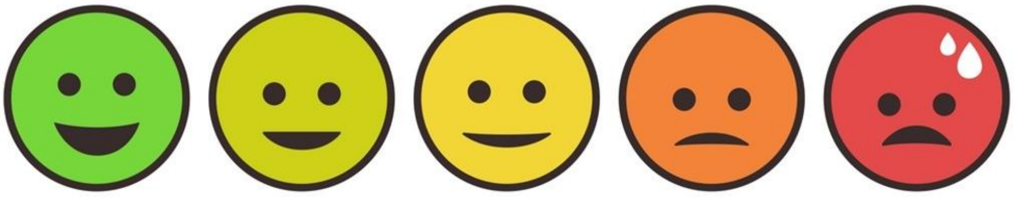

Food security

Please answer these about the last year

31. We can afford to eat properly.

(*i.e., whether you think your household has enough money to eat properly*)

- ☐ Often
- ☐ Sometimes
- ☐ Never
- ☐ Don't know
- ☐ Prefer not to say

32. Food runs out in our household due to lack of money.

(*i.e., whether you run out of basics like bread, potatoes, not treats of special foods*)

- ☐ Often
- ☐ Sometimes
- ☐ Never
- ☐ Don't know
- ☐ Prefer not to say

33. We eat less because of lack of money.

(*i.e., whether you have smaller meals, skip meals or don't have seconds because of lack of money*)

- ☐ Often
- ☐ Sometimes
- ☐ Never
- ☐ Don't know
- ☐ Prefer not to say

34. The variety of foods we are able to eat is limited by a lack of money.

(*i.e., whether you don't eat many different kinds of foods because of lack of money*)

- ☐ Often
- ☐ Sometimes
- ☐ Never
- ☐ Don't know
- ☐ Prefer not to say

35. We rely on others to provide food and/or money for food when we do not have enough money for food.

(*i.e., whether you rely on support and assistance from other people for food*)

- ☐ Often
- ☐ Sometimes
- ☐ Never
- ☐ Don't know
- ☐ Prefer not to say

36. We make use of special food grants or food banks when we do not have enough money for food.
- Often
- Sometimes
- Never
- Don’t know
- Prefer not to say

37. I feel stressed because of not having enough money for food.
- Often
- Sometimes
- Never
- Don’t know
- Prefer not to say

38. I feel stressed because I can’t provide the food I want for social occasions.
(i.e., whether you feel stressed or whakamā because you don’t have enough to share food with others of give koha of food)
- Often
- Sometimes
- Never
- Don’t know
- Prefer not to say

About You

39. My age is

- ☐ 15-19 years
- ☐ 20-24 years
- ☐ 25-44 years
- ☐ 45-64 years
- ☐ 65+ years
- ☐ Prefer not to say

40. I identify as

- ☐ Female
- ☐ Male
- ☐ Another gender, please specify if comfortable doing so ……………………
- ☐ Prefer not to say

41. My ethnicity is (choose as many as apply)

- ☐ Māori
- ☐ New Zealand European
- ☐ Samoan
- ☐ Cook Island Maori
- ☐ Tongan
- ☐ Niuean
- ☐ Chinese
- ☐ Indian
- ☐ Another ethnicity, please specify if comfortable doing so ……………………
- ☐ Prefer not to say

42. How many adults (18 or over) are there in your household? _______

43. How many children (under 18) are there in your household? _______

44. How do you rate your health?

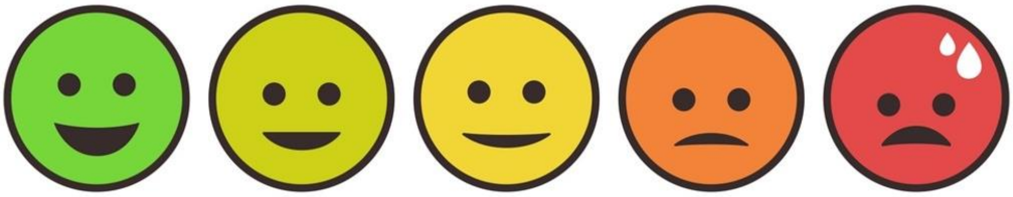

End of survey. Ka nui te mihi ki a koe. Thank you for your time.
